# Fate and transport of urea-N in a rain-fed ridge-furrow crop system with plastic mulch

**DOI:** 10.1016/j.still.2018.10.022

**Published:** 2019-03

**Authors:** Sheng Guo, Rui Jiang, Hongchao Qu, Yilin Wang, Tom Misselbrook, Anna Gunina, Yakov Kuzyakov

**Affiliations:** a Key Laboratory of Plant Nutrition and the Agri-environment in Northwest China, Ministry of Agriculture, College of Natural Resources and Environment, Northwest A&F University, Yangling, 712100, China; b Department of Sustainable Agricultural Sciences, Rothamsted Research, North Wyke, Okehampton, Devon, EX20 2SB, UK; c Department of Agricultural Soil Science, Georg-August-University of Göttingen, Göttingen, 37077, Germany; d Institute of Environmental Sciences, Kazan Federal University, 420049, Kazan, Russia; e Agro-Technology Institute, RUDN University, Moscow, Russia; f Department of Soil Biology and Biochemistry, Dokuchaev Soil Science Institute, Russia

**Keywords:** Loess plateau, ^15^N labeling, Plant N uptake, Soil residual N, N losses

## Abstract

A better understanding of the fate and transport of fertilizer nitrogen (N) is critical to maximize crop yields and minimize negative environmental impacts. Plastic film mulching is widely used in drylands to increase soil water use efficiency and crop yields, but the effects on fertilizer N use efficiency need to be evaluated. A field experiment with ^15^N-urea (260 kg N ha^−1^) was conducted to determine the fate and transport of fertilizer N in a ridge-furrow system with plastic film mulched ridge (Plastic), compared with a flat system without mulching (Open). In the Plastic, the ^15^N-urea was applied to the ridge only (Plastic-Ridge), or to the furrow only (Plastic-Furrow). Maize grain yield and net economic benefit for Plastic were significantly higher (by 9.7 and 8.5%, respectively) than those for Open. Total plant ^15^N uptake was 72.5% greater in Plastic compared with Open, and ^15^N was allocated mostly to the grain. Losses of the applied urea-N were 54.5% lower in Plastic and much more residual ^15^N was recovered in 0–120 cm soil compared with Open (42.7 and 26.8% of applied ^15^N, respectively). Lateral N movements from furrow to ridge and from ridge to furrow were observed and attributed to lateral movement of soil water due to microtopography of ridges and furrows and uneven soil water and heat conditions under mulching and plant water uptake. The ridges were the main N fertilizer source for plant uptake (96.5 and 3.5% of total N uptake in Plastic from ridge and furrow, respectively) and the furrow was the main source of N losses (78.6 and 21.4% of total N losses in Plastic from furrow and ridge, respectively). Gas emissions, especially ammonia volatilization was probably the main N loss in furrow. Thus, appropriately localized N application – into the ridges, and management strategies should be designed for Plastic to maximize N use efficiency by crops, decrease N gas losses and maintain sustainable agricultural systems in drylands.

## Highlights

• Plastic increased total plant ^15^N uptake rather than Open.
• More residual ^15^N was recovered in 0–120 cm soil under Plastic than under Open.
• The ridge was the main N fertilizer source for plant uptake.
• Lateral N movements from furrow to ridge and from ridge to furrow were observed.

## 1 Introduction

To meet the needs of a growing population and ensure food security, large quantities of N are applied to farmland to achieve high yields (Yang et al., 2015; Abbasi et al., 2012). Excessive fertilization, however, results in N losses and environment pollution (Granlund et al., 2008). Maximizing crops yields while minimizing negative environmental impacts is one of the major current challenges in agriculture (Li et al., 2011; Yang et al., 2015).

Plastic film mulching was introduced in China in 1978, originally only for vegetables but now is widely used for maize, wheat, potato and other staple crops (Dong et al., 2009). Plastic film mulching increases crop yield, especially in arid and semi-arid areas, due to higher soil temperature, less water losses and consequently higher soil moisture, and higher nutrient availability (Wang et al., 2004; Bu et al., 2013; Chakraborty et al., 2008; Liu et al., 2015). Plastic film mulching increases soil temperature due to the "greenhouse effect", which plays an important role in the early growth stage of crops (Gan et al., 2013). Soil moisture under mulching is increased by collecting light rain, strongly reducing evaporation, and promoting rainfall infiltration (Wang et al., 2009; Zegada-Lizarazu and Berliner (2011). These soil conditions may influence the fate of applied N fertilizers through enhancing plant N uptake and N use efficiency and reducing the risk of nitrate leaching (Ruidisch et al., 2013; Zhou et al., 2012). However, there is still no a clear concepts and experiments how plastic mulching affects the fate of N fertilizer in soils.

Plastic film mulching increases crop yield, plant N uptake (Liu et al., 2014a) and N use efficiency and decreases N losses (Liu et al., 2015) including yield-scaled N_2_O emission (Liu et al., 2014a). Higher grain yields and N recovery, and lower N losses from a plastic mulched maize cropping system were obtained in a semiarid region with two split N applications (Wang et al., 2016a). N mineralization increased under plastic film mulching, but nitrate leaching also increased if mulching was in place for the whole growing season (Zhang et al., 2012). Management of the mulch duration is therefore necessary to achieve a balance between N mineralization and leaching (Zhang et al., 2012). Combined with effective N management practices, plastic film mulching has the potential to improve sustainability and confer economic and environmental benefits (Romic et al., 2003; Wang et al., 2016b).

Several plastic film mulching systems have become popular in recent years. Among them, the ridge-furrow system with plastic film mulched ridges (Plastic) has been one of the most effective cultivations to increase water use efficiency, soil temperature and yields for rain-fed croplands in Northwestern China, especially in the region with 400–600 mm of precipitation (Gan et al., 2013). This Plastic system contains plastic film covered ridges and uncovered furrows (Fig. 2). The uneven soil water and heat conditions caused by plastic film mulching and the soil microtopography caused by ridges and furrows may be different from other plastic film mulching systems. The soil water and temperature differ between ridges and furrows (Jiang et al., 2016). The Plastic system enhances rainwater harvest in the furrow and increases rainwater infiltration (Wang et al., 2009), which may increase N leaching and reduce N uptake from furrow. The plastic film covering the ridge may decrease N leaching and increase the plant N uptake from the ridge. The differences in soil water and temperature conditions between ridges and furrows cause lateral movement of water (Jiang et al., 2016; Ruidisch et al., 2013b), which may lead to lateral N transport and redistribution of residual soil N. Consequently, the non-uniform distribution of water may affect the utilization and redistribution of fertilizer N. With such heterogeneity, the furrows and ridges should be treated as two management units (Kettering et al., 2013) and N fertilization should be site specific. Kettering et al. (2013) showed that, under a monsoon climate, nitrate leaching mainly occurred through the furrows in the Plastic system.

We hypothesize that plant N uptake and N losses will differ between the furrows and ridges in the Plastic system, and will have various advantages compared to the soil without plastic mulching. There are very few published data focused on this comparison and, in particular, no studies have simultaneously measured both lateral and vertical N transport. Therefore, we investigated the fate of fertilizer N for maize production in a semi-arid area using a ^15^N tracer technique in the Plastic system, compared with a flat system without mulching (Open), to examine (1) plant N uptake and N losses; (2) vertical and lateral redistribution of residual N in soil; (3) differences in the fate of applied N for ridges and furrows.

## 2 Materials and methods

### 2.1 Study site

**Fig. 1:**
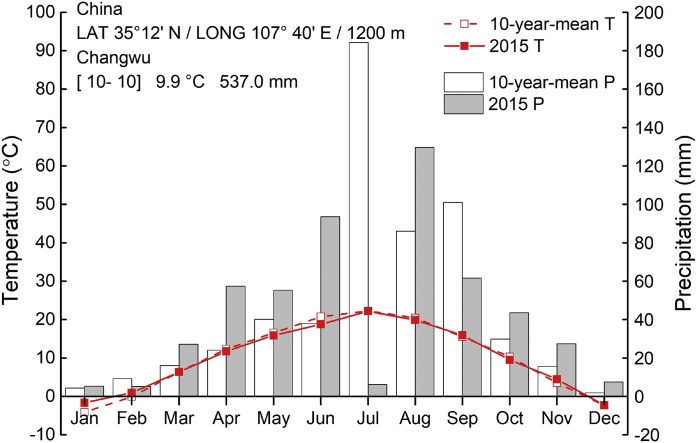
Monthly mean precipitation and average temperature (10 years average from 2005 to 2014 and year 2015 only).

The study site was located on the Loess Plateau at the Changwu Agricultural and Experimental Station of the Chinese Academy of Sciences (latitude 35˚12′N, longitude 107˚40′E, elevation 1200 m asl) and has a semi-arid climate. Mean annual air temperature is 9.1 ℃; the average annual precipitation is 580 mm and more than 60% of total precipitation falls in July, August, and September (Fig. 1). The average potential evaporation is 1560 mm. The depth of groundwater is 50–80 m. The frost-free season lasts 171 days. The soil at the experimental site has a silt loamy texture according to the USDA texture classification system. The physico-chemical soil properties in the top 20 cm are given in Table 1.

**Fig. 2:**
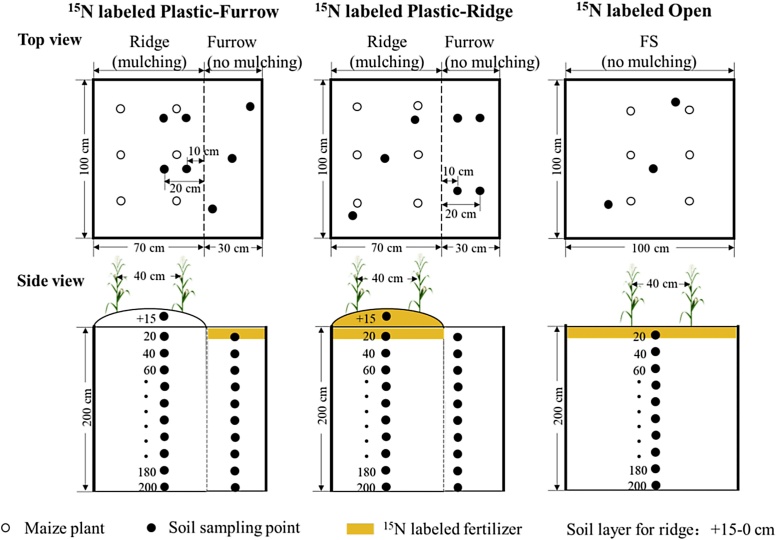
Top and side views of ^15^N labeled Plastic-Ridge, Plastic-Furrow, and Open in micro-plots (Open: the flat system without mulching; Plastic: the ridge-furrow system with plastic film mulched ridges; Plastic-Ridge: the ^15^N-labeled fertilizer in Plastic was applied to the ridges only; and Plastic-Furrow: the ^15^N-labeled fertilizer in Plastic was applied to the furrows only).

**Table 1:** Physico-chemical properties of the soil.

Soil depth (cm)	pH	Bulk density (g cm^−3^)	Total N (g kg^−1^)	Organic C (g kg^−1^)	Total P (g kg^−1^)	Total K (g kg^−1^)	Mineral N (mg kg^−1^)
0–20	8.4 ± 0.2	1.17 ± 0.10	1.02 ± 0.14	7.8 ± 1.9	0.97 ± 0.27	11.50 ± 1.24	28.3 ± 2.35

### 2.2 Experimental design

Two cropping systems were included (Fig. 2): (1) a flat system without mulching (Open); (2) a ridge-furrow system with plastic film mulched ridge (Plastic). Each cropping system was replicated four times in a randomized block arrangement and each plot was 45 m^2^ (4.5 × 10 m). The urea application rates were the same in all plots: 260 kg N ha^−1^ (local N application rate), 40 kg P ha^−1^, and 75 kg K ha^−1^. All fertilizers were spread over the plots and mixed with the 0–15 cm surface soil by rake as a basal dressing, as is the current farming practice. Ridges and furrows were made after fertilizer application in Plastic. The Plastic comprised alternative furrows (30 cm wide) and ridges (15 cm high × 70 cm wide), and the ridges were covered with plastic film (0.008 mm thick and 90 cm wide). Maize was planted on each shoulder of the ridge with a spacing of 30 cm along the ridge, 40 cm across the ridge and 60 cm across the furrow (Jiang et al., 2016). The Open also had identical wide (60 cm) and narrow (40 cm) row spacing (Jiang et al., 2016). A high-yielding maize hybrid (Pioneer 335) was hand planted at a density of 66,667 plants ha^−1^ on 30 April 2015 and harvested on 27 September 2015. The precipitation in this period was 346 mm (Fig. 1), and no irrigation was used. After maize harvest, the aboveground biomass was removed and the plastic film mulching was left in the Plastic until the next crop sowing.

To study the fate of the applied fertilizer N, micro-plots (1 × 1 m, 1 m^2^, covering a complete ridge and furrow, including 6 plants, Fig. 2) were established in the center of the plots. The top soil (0–15 cm) was removed from the micro-plot, passed through a 2 mm sieve and mixed with ^15^N-labeled urea (10.0 atom% ^15^N, provided by Shanghai Research Institute of Chemical Industry) and the P and K fertilizers, then the soil and fertilizers were returned to the micro-plot. In Plastic, the ^15^N-labeled fertilizer was applied in two positions in the separated micro-plots: 1) applied to the ridge only (Plastic-Ridge) and 2) applied to the furrow only (Plastic-Furrow). The fertilizer application rate for ridges and furrows in the micro-plots was the same as for the large plots, with the ridge: furrow ratio of 7:3 (based on pre-experiment in 2014: measurements of total N for ridge and furrow in the Plastic before and three weeks after N fertilizer applied in this study plot and on local famers' fields in 2014). In Open, the ^15^N-labeled fertilizer was applied evenly across the whole surface layer of the micro-plot. All micro-plots were enclosed by an aluminum sheet inserted into the soil to a depth of 2 cm and exposed 3 cm above the surface to prevent surface runoff.

### 2.3 Soil and plant sampling and analysis

At harvest in 2015, six ^15^N-labeled plants in each micro-plot were harvested and separated into grain, leaf, stem, cob core and bract. Part of roots during harvest was decomposed due to a rainfall, thus the main root part was grouped into stem and the decomposed part was remained in soil. Dry weight was determined by drying at 105 ℃ for 30 min and then at 70 ℃ to constant weight. Soil samples were collected from three points per plot from 0 to 200 cm in 20 cm layers and also from the ridge (+15-0 cm soil layer). Soil samples were taken at the same depths in the micro-plots (Fig. 2). Three points in the ^15^N-labeled ridge were taken and mixed into one sample for each depth, and the same for the ^15^N-labeled furrow. To assess lateral movement of N, two points located at 10 cm and a further two points at 20 cm laterally from the labeled ridge in the unlabeled furrow in Plastic-Ridge were also taken; and similarly for Plastic-Furrow. The duplicate samples (at 10 cm or 20 cm from labeled ridge/furrow) were combined as a single sample for each depth (Fig. 2).

The content of NO_3_^−^ and NH_4_^+^ in fresh soil were extracted by 2 M KCl and determined using a Continuous Flow Analyzer (AA3, Seal, Germany). Mineral N was calculated as the sum of NO_3_^−^ and NH_4_^+^. The content of microbial biomass N in soil was measured by the chloroform fumigation-extraction method Brookes et al. (1985). Soil water content was measured gravimetrically. Air-dried soil samples were ground and passed through a 1.5 mm sieve for analysis for total N. Total N content in plant and soil samples was analyzed using the Kjeldahl method (Bremner and Mulvaney, 1982).

The ^15^N abundance in soil and plant samples was determined by isotope ratio mass spectrometer (IRMS) at UC Davis Stable Isotope Facility, University of California. The ammonium diffusion method (Brooks et al., 1989) followed by IRMS was used to determine the mineral ^15^N abundance. The chloroform fumigation-extraction method combined with ammonium diffusion method was used to determine microbial biomass ^15^N (MBN). The natural abundance of ^15^N in soil and plant samples was also measured.

### 2.4 Calculation and statistical analyses

Plant total N uptake (N_pu_, kg ha^−1^), plant N derived from ^15^N-labeled fertilizer (N_dff_, kg ha^−1^), plant N derived from soil (N_dfs_, kg ha^−1^), fertilizer N recovery (N_rec_, %), soil residual N (N_resid_, kg ha^−1^) and N fertilizer lost to the environment (N_loss_, kg ha^−1^) were calculated according to Eqs. (1) to (6) below, respectively.

(1) $$N_{pu}={DM}_{p}\times N_{p}$$

where DM_p_ is plant dry matter yield (kg ha^−1^) and N_p_ is plant N content (kg kg^−1^) dry matter;

(2) $$N_{dff}=N_{pu}\times {{\;}^{15}N_{\;}\%}_{p}/{\;}^{15}N_{\;}{\%}_{f}$$

where ^15^N%_p_ and ^15^N%_f_ are the atom% ^15^N excess in the plant and soil, respectively;

(3) $$N_{dfs}=N_{pu}-N_{dff}$$

(4) $$N_{rec}=\left(N_{pu}/N_{rate}\right)\times 100$$

where N_rate_ is the fertilizer N application rate (kg ha^−1^);

(5) $$N_{resid}={{\;}^{15}N_{\;}}_{soil}\times {soil}_{bd}\times d\times 10,000$$

where ^15^N_soil_ is the soil ^15^N content (g kg^−1^ dry soil), soil_bd_ is the soil bulk density (g cm^-3^) and d the depth of soil sampled (m);

(6) $$N_{loss}=N_{rate}-N_{pu}-N_{resid}$$

Cost-benefit analysis included assessment of the total costs, income from grain sales and net economic benefit (NEB). The total costs included the cost of field operations (labor cost associated with fertilizer/pesticide applications and mechanical operations), fertilizer/pesticide/seed, and plastic film (http://www.npcs.gov.cn/ and http://china.guidechem.com/). Income refers to income from grain yield. The NEB was calculated by subtracting the input cost from the yield income (Ma et al., 2018).

The paired sample *t*-test was used and least significant differences (*p* < 0.05) were calculated to test for significance of differences in grain yield, dry matter biomass, N_pu_, N_dff_, and N_dfs_ between Open and Plastic. The differences between treatments (Plastic-ridge, Plastic-furrow, and Open) were tested with one-way analysis of variance (ANOVA) and following Tukey-test (*p* < 0.05). The differences between depths we tested with one sample *t*-test. Homogeneity of variances was tested by Levene’s test, normal distribution of residues was tested by Shapiro test. All the data analyses were performed using SPSS 22. Graphs were produced with Origin 9.1.

## 3 Results

### 3.1 Plant biomass, N uptake, and 15N distribution in maize

The grain yield was 9.7% higher in Plastic than that in Open (*p* < 0.05), but stem, cob cores, bract and total aboveground biomass were similar (Table 2). Plastic increased total N uptake by 13.6% (*p* < 0.05), compared with Open, especially in the grain and stems. The plant N uptake derived from the soil (N_dfs_) was similar for the two systems (*p* > 0.05), accounting for 71.3% and 80.6% of plant N uptake for Plastic and Open, respectively. In contrast, the plant N uptake derived from urea-fertilizer (N_dff_) was 71.1% higher for Plastic than for Open (*p* < 0.05, Table 2).

**Table 2:** Effect of plastic film mulching on maize plant biomass, total N uptake, N uptake derived from soil (Ndfs), and N uptake derived from ^15^N-labeled fertilizer (Ndff) in plant parts. Superscript letters within a column indicate a significant differences (*p* < 0.05) between Plastic and Open (Plastic: the ridge-furrow system with plastic film mulched ridge; Open: the flat system without mulching).

Plant parts	Cropping systems	Dry matter (Mg ha^−1^)	Total N uptake (kg N ha^−1^)	N_dfs_ (kg N ha^−1^)	N_dff_ (kg N ha^−1^)
Grain	Plastic	14.2 ± 0.2^a^	201 ± 5^a^	143 ± 4^a^	58.4 ± 1.6^a^
	Open	12.9 ± 0.4^b^	174 ± 3^b^	144 ± 3^a^	30.0 ± 0.5^b^
Leaf	Plastic	3.5 ± 0.7^a^	51.9 ± 5.6^a^	37.1 ± 4.0^a^	14.8 ± 1.6^a^
	Open	3.5 ± 0.2^a^	52.9 ± 1.1^a^	39.9 ± 0.8^a^	13.0 ± 0.3^a^
Stem	Plastic	8.3 ± 1.1^a^	24.3 ± 4.5^a^	18.8 ± 3.5^a^	5.5 ± 1.0^a^
	Open	7.1 ± 0.4^a^	12.4 ± 0.3^b^	9.3 ± 0.2^b^	3.1 ± 0.1^b^
Cob core	Plastic	1.9 ± 0.0^a^	10.9 ± 3.4^a^	6.5 ± 2.0^a^	4.4 ± 1.4^a^
	Open	1.9 ± 0.0^a^	7.4 ± 1.2^b^	5.6 ± 0.9^b^	1.8 ± 0.3^a^
Bract	Plastic	3.1 ± 1.4^a^	8.5 ± 0.9^a^	6.3 ± 0.7^a^	2.2 ± 0.2^a^
	Open	2.9 ± 0.1^a^	9.4 ± 0.5^a^	7.5 ± 0.4^a^	1.9 ± 0.1^a^
Aboveground plant parts	Plastic	31.0 ± 3.5^a^	297 ± 20^a^	211 ± 14^a^	85.2 ± 5.7^a^
	Open	28.3 ± 1.1^a^	256 ± 6^b^	207 ± 5^a^	49.8 ± 1.2^b^

**Fig. 3:**
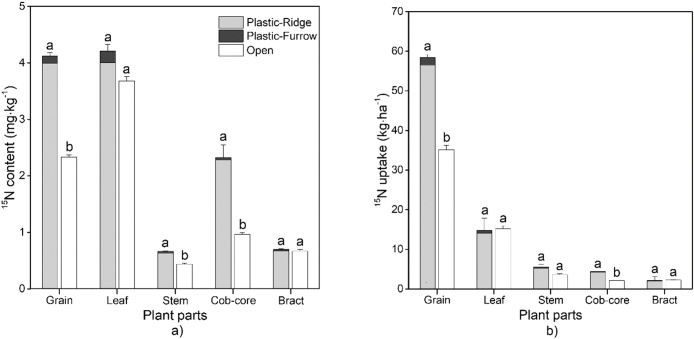
Effects of plastic film mulching on distribution of a) ^15^N content and b) ^15^N uptake among maize plant parts at harvest in 2015 (Open: the flat system without mulching; Plastic: the ridge-furrow system with plastic film mulched ridge; Plastic-Ridge: the ^15^N-labeled fertilizer in Plastic was applied to the ridge only; and Plastic-Furrow: the ^15^N-labeled fertilizer in Plastic was applied to the furrow only).

The ^15^N content and ^15^N uptake in various aboveground plant parts showed the same trends: Ridge > Open > Furrow, although not always significantly different (Fig. 3). Among the plant parts, ^15^N content was the highest in grains and leaves in Plastic, while the highest was in leaves in Open. (Fig. 3). The proportion of ^15^N uptake from the ridge was much higher than that from the furrow. The ^15^N uptake in grain was the highest among all plant parts, accounting for 68.5 and 60.2% of the total plant ^15^N uptake in Plastic and Open, respectively. This was followed by uptake in leaves, accounting for 17.4 and 26.1%, respectively, and the uptake of ^15^N in the other plant parts was only 14.1 and 13.7%, respectively (Fig. 3, Table 2).

### 3.2 Redistribution of residual ^15^N in soil

#### 3.2.1 Vertical distribution of residual ^15^N in soil

**Fig. 4:**
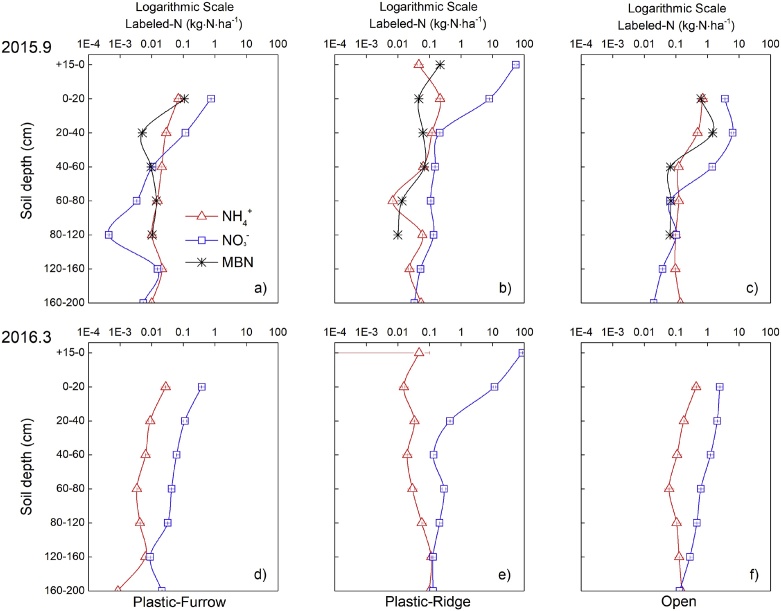
Distribution of residual mineral ^15^N (NH_4_^+^ and NO_3_^−^) and microbial biomass ^15^N (MBN) in the 0–200 cm soil layer in Plastic-Furrow, Plastic-Ridge and Open at harvest in September 2015 (a–c) and before sowing in March 2016 (d–f) (Open: the flat system without mulching; Plastic: the ridge-furrow system with plastic film mulched ridges; Plastic-Ridge: the ^15^N-labeled fertilizer in Plastic was applied to the ridges only; and Plastic-Furrow: the ^15^N-labeled fertilizer in Plastic was applied to the furrows only).

The mineral-^15^N (the sum of NH_4_^+^-^15^N and NO_3_^−^-^15^N) accounted for 19.0–87.3% of total residual ^15^N in the soil layers for Plastic-Ridge at harvest in 2015, with an average of 48.2%. Respective values were 7.5–26.1% (average 15.1%) and 8.3–52.6% (average 29.6%) for Plastic-Furrow and Open, respectively. The NH_4_^+^-^15^N content was lower than NO_3_^−^-^15^N in all soil layers. The soil mineral ^15^N was higher in Plastic than that in Open (*p* < 0.05). Microbial biomass ^15^N was low, accounting for 0.3–12.4% of the total residual ^15^N in Plastic-Ridge, and 0.3–19.6% and 1.2–11.1% in Plastic-Furrow and Open, respectively. The larger ^15^N incorporation in microbial biomass occurred at soil depths of +15-0 cm, 0–20 cm, and 0–40 cm in Plastic-Ridge, Plastic-Furrow and Open, respectively (Fig. 4a–c). The microbial biomass ^15^N was larger in Open than in Plastic (*p* < 0.05).

The depth distribution of residual NH_4_^+^-^15^N and NO_3_^−^-^15^N in the soil were similar in Plastic-Furrow, Plastic-Ridge and Open at harvest (Fig. 4a-c). The residual ^15^N decreased gradually with depth. More than 90% of the mineral ^15^N in the 0–200 cm soil profile was distributed in the upper 20 cm soil layer in Plastic-Ridge, with very little below 40 cm. In Plastic-Furrow, the residual mineral ^15^N was mainly distributed in 0–60 cm soil layer. In Open, more of the residual mineral ^15^N was distributed in the deeper soil layers, with 84.9% in the upper 40 cm, and 12.1% in the 40–60 cm soil layer. The total soil mineral ^15^N was 10–100 times higher in Plastic-Ridge than in Open or Plastic-Furrow.

Compared with the 2015 harvest, the residual mineral ^15^N in the soil before sowing in 2016 had moved vertically from the surface to the deeper soil layers (Fig. 4). In the Plastic-Furrow and Open, mineral-^15^N at 0–20 cm soil layer decreased by 50.5% and 32.3%, respectively. However, the mineral-^15^N in the top soil layers (+15-20 cm) in Plastic-Ridge was 58.9% higher than that at harvest in 2015 (Fig. 4). Across the whole soil profile (0–200 cm), the residual mineral ^15^N decreased by 34.0% and 40.4% in Plastic-Furrow and Open, but increased by 59.6% in Plastic-Ridge between harvest in 2015 and sowing in 2016.

**Fig. 5:**
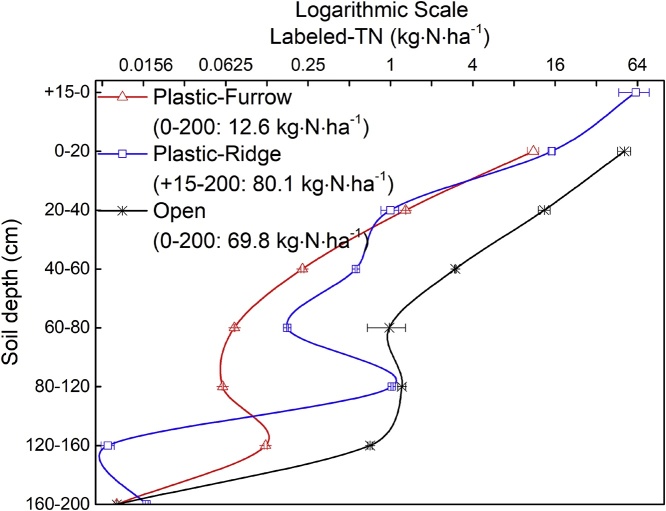
Vertical distributions of residual total ^15^N in 0–200 cm soil layer depending on cropping systems at harvest in 2015 (Open: the flat system without mulching; Plastic: the ridge-furrow system with plastic film mulched ridges; Plastic-Ridge: the ^15^N-labeled fertilizer in Plastic was applied to the ridges only; and Plastic-Furrow: the ^15^N-labeled fertilizer in Plastic was applied to the furrows only).

The distribution of total residual ^15^N in soil was similar to that of the mineral-^15^N, i.e. most of the ^15^N was located in the top 20 cm soil layer. At harvest in 2015, the total residual ^15^N in the upper 20 cm layer was 11.1, 77.4 and 51.3 kg ha^−1^ in the Plastic-Furrow, Plastic-Ridge and Open soil, respectively, accounting for 87.0, 96.6 and 73.5% of the total residual ^15^N in the 0–200 cm. Most of total ^15^N was in the ridge (+15-0 cm) for Plastic, while for Open it was mostly in the 0–60 cm soil layers (Fig. 5).

#### 3.2.2 Lateral distribution of residual ^15^N in soil

**Fig. 6:**
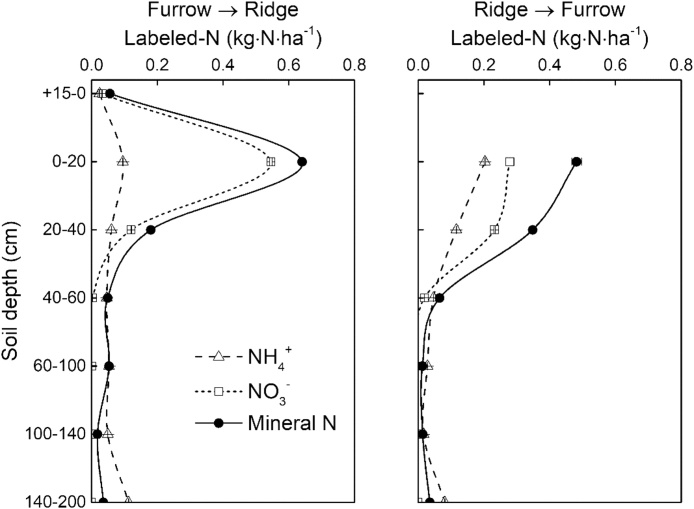
The lateral distribution of residual mineral ^15^N in 0–200 cm in Plastic-Furrow and Plastic-Ridge at harvest in 2015 (Furrow → Ridge: ^15^N moved from furrow to ridge in Plastic-Furrow; Ridge → Furrow: ^15^N moved from ridges to furrows in Plastic-Ridge; Plastic-Ridge: the ^15^N-labeled fertilizer in Plastic was applied to the ridges only; and Plastic-Furrow: the ^15^N-labeled fertilizer in Plastic was applied to the furrows only).

**Fig. 7:**
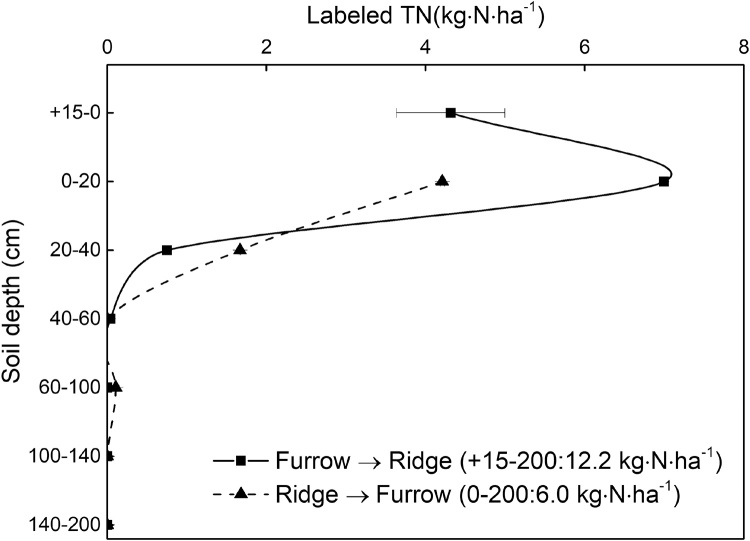
Lateral movement of total residual ^15^N in the 0–200 cm soil profile for Plastic-Furrow and Plastic-Ridge at the 2015 harvest (Furrow → Ridge: ^15^N moved from furrows to ridges in Plastic-Furrow; Ridge → Furrow: ^15^N moved from ridges to furrows in Plastic-Ridge).

The residual ^15^N in Plastic-Furrow was not only observed in the ^15^N-labeled furrow, but also in the upper 40 cm soil layer in an unlabeled ridge at distances of 10 and 20 cm from the ^15^N-labeled furrow, with 4.3 and 7.0 kg ha^−1^ of the fertilizer ^15^N in the +15-0 and 0–20 cm soil layers in the ridge. Similarly, ^15^N from the fertilizer applied to the Plastic-Ridge was observed in the upper 40 cm soil layer in an unlabeled furrow at distances of 10 and 20 cm from the ^15^N-labled ridge, with most (4.2 kg ha^−1^) in the 0–20 cm soil layer (Fig. 6, Fig. 7). This clearly indicates that lateral movement of N between ridges and furrows. Total amounts of fertilizer ^15^N that moved from furrow to ridge (Furrow → Ridge) and from ridge to furrow (Ridge → Furrow) were 12.2 and 6.0 kg ha^−1^, respectively (Fig. 7). The lateral movement of N was probably related to soil water movement due to the microtopography of ridges and furrows, uneven soil water and heat conditions under mulching and plant water uptake. However, mineral ^15^N only accounted for 16.0 and 8.6% of the total amount of ^15^N movement for Ridge → Furrow and Furrow → Ridge, respectively, indicating that after the lateral N movement, N was immobilized by microorganisms or clay minerals.

### 3.3 Fate of ^15^N-urea in Plastic and Open systems

**Fig. 8:**
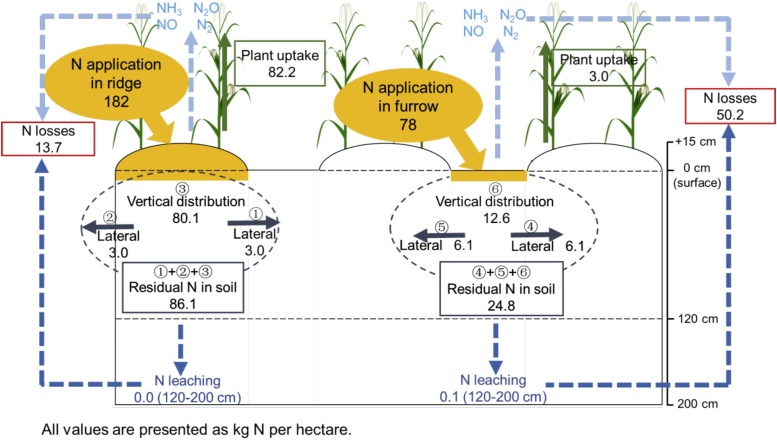
Distribution of fertilizer N (^15^N) in the 0–200 cm soil profile for the Plastic-Furrow and Plastic-Ridge components of the Plastic system at harvest in 2015 (all values are presented as kg N ha^−1^).

Compared with Open, total ^15^N uptake in the aboveground maize biomass increased under the Plastic system, with an associated decrease in ^15^N losses (Table 3). In Plastic, plant ^15^N uptake and residual ^15^N in the soil were 71.1 and 58.8% higher than that in Open. Plastic decreased the N loss by 54.5%, compared with Open. N leaching was very low, both in Plastic and Open. The recovery and potential losses in Plastic were 3.8 and 64.4%, respectively, for Plastic-Furrow, and 45.2 and 7.5% for Plastic-Ridge, respectively. The 96.5% of total plant ^15^N uptake derived from the ridge in Plastic, and only 3.5% from the furrow (Fig. 8). Redistribution of residual soil ^15^N included the vertical and lateral distribution components in Plastic-Ridge and Plastic-Furrow. The Ridge → Furrow movement accounted for 7.0% of residual soil ^15^N in Plastic-Ridge, and Furrow → Ridge movement accounted for 49.2% of residual soil ^15^N in Plastic-Furrow.

**Table 3:** The fate of fertilizer ^15^N depending on the mulching system and initial fertilizer application locations.

Cropping systems	N application rate (kg ha^−1^)	Plant N uptake (kg ha^−1^)	Recovery (%)	Residual N in soil (kg ha^−1^)	Residual (%)	Potential N Loss (kg ha^−1^)		Loss (%)
						N leaching*	Potential gas emissions^*^	
Plastic	260	85.2	32.8	110.9	42.7	0.1	63.8	24.5
Open	260	49.8	19.2	69.8	26.8	0.7	139.6	54.0

* note: N leaching was measured as the total ^15^N in the soil layers of 1.2–2 m. N distributed in the soil depth below 1.2 m was considered as potentially leached, because there were not any roots of maize below this depth. Although nitrate may have also leached beyond 2 m, the amount was considered to be very small because of the low soil ^15^N content (close to 0) at 120–200 cm (Fig. 4); Potential gas emissions were the calculated by ^15^N balance.

### 3.4 N balance and net economic benefits

The total N input (including N from fertilizer application, non-symbiotic N fixation, deposition and seed) was the same (297 kg N ha^−1^ yr^−1^) for Plastic and Open, but the surplus N was different due to higher crop uptake and lower potential N loss in Plastic (Table 4). The cost and economic benefit were also calculated. Although there were extra costs including the capital cost of the plastic film cost and the operational cost of forming the ridges and applying the plastic mulch in Plastic, the net economic benefit increased by 8.5%, compared with Open (Table 5).

**Table 4:** The N balance (kg N ha^–1^ yr^–1^) in different cropping systems.

	Plastic	Open
Input		
Fertilizer N	260	260
Non-symbiotic N fixation ^a^	15	15
Deposition ^b^	21	21
Seed	1	1
Total	297	297
Output		
Crop uptake	297	256
Potential N loss ^c^	64	140
Surplus	−64	−99

a) The non-symbiotic N fixation in the study area was 15 kg N ha^–1^ yr^–1^ (Ju et al., 2017); b) the N deposition in the study area was 21 kg N ha^–1^ yr^–1^ (Wang et al., 2008); c) the potential N losses estimate is that from the fertilizer, based on the N budget using the isotope ^15^N method.

**Table 5:** The costs and economic benefits ($ ha^–1^ yr^–1^) for maize production depending on management.

	Plastic	Open
Costs		
Plastic film	68	–
Field operations	76	55
Fertilizer/pesticide/seed	316	316
Total cost	460	371
Income		
Grain	4135	3756
Net economic benefit	3675	3385

## 4 Discussion

### 4.1 Effects of plastic mulch on grain yields, biomass and N uptake

Although total aboveground biomass was similar for the Plastic and Open, the grain yield and grain N content were significantly greater for Plastic, by 9.7 and 5.1%, respectively (Fig. 3, Table 2), indicating the increase of N transfer to grain under the Plastic system. Increased grain yields under the Plastic system have been widely reported (Bu et al., 2013). The mechanisms of yield increases include: 1) higher water availability because of reduced evaporation during drought periods under plastic film mulching; 2) an increase in the soil temperature what is especially important in the early stage of the maize growing season, enhancing crop growth (Jiang et al., 2018; Ramakrishna et al., 2006; Zhao et al., 2012). Liu et al. (2015b) found that the grain yields and aboveground biomass under plastic film mulching were 70 and 53% higher than that without mulching, respectively. The lack of a difference in aboveground biomass and an increase of only 9.7% in grain yield in this study was most likely because of the wetter than usual conditions during the early stage of the maize growing season (the amount of rainfall during early stage of maize growing season in 2015, April to June, was 50 mm lager than the 30 year average, Fig. 1). The early stage of maize growth season usually suffers drought and is crucial for maize production (Jiang et al., 2016). The mechanism of plastic film mulching in yield increase was mostly because mulching changed the soil water and temperature condition during drought period at early stage of maize growing season (Jiang et al., 2016, 2018). Thus the better conditions for maize growth at early stage in 2015 mean that the benefit of plastic film mulching in yield increase was not as large as in other studies. The plastic film mulching increased the proportion of total plant ^15^N uptake in grain (Fig. 3). A 15.7% higher total N uptake was observed in Plastic duo to higher N uptake in grain, compared with Open. Similar higher N uptake of 14–34% was reported in the southern part of the Loess Plateau (Wang et al., 2014), in Gwalior (Bhadauria et al., 2015), and in southern Nigeria (Mbagwu et al., 2010). In our study, 19.4–28.7% of plant N uptake was derived from the applied fertilizer in Plastic and Open (Table 2). These results suggest that mineralization of soil organic matter is the main source of N uptake in maize, which is consistent with Wang et al. (2016a) and Rimski-Korsakov et al. (2012).

### 4.2 Effects of plastic mulch on the fate of urea-N

The plastic film mulching affected the fate of the applied N fertilizer: decreased N losses and increased the plant N uptake and residual soil N compared to Open (Table 3). This may be explained by lower ammonia volatilization under plastic film mulching and therefore, more fertilizer N remaining in the soil available for plant uptake (Liu et al., 2015; Wang et al., 2016a). Additionally, the immobilization of urea-^15^N by microorganisms may occur at an early stage in the maize growing season due to the increased microbial activity under the mulched soil. Subsequent slow mineralization of the microbially-immobilized organic N throughout the growing season may lead to enhanced uptake and utilization of the fertilizer N by the maize plants. This is the common temporal niche partitioning between plants and microorganisms to decrease competition for N (Kuzyakov and Xu, 2013). This hypothesis needs further testing, particularly through measurements of N partitioning between plants and microorganisms. However, Liu et al. (2015b) found that mulching decreased fertilizer N recovery by maize, and attributed it to a "dilution effect" of increased soil N availability due to the increased N mineralization from soil organic matter compared with that in soil without mulching. Therefore, the plant ^15^N uptake in Plastic also depends on N mineralization-immobilization and applied fertilizer N (Jenkinson et al., 2010; Kuzyakov et al., 2000).

The 42.7% of applied ^15^N remained in the 0–120 cm soil layer for Plastic (Table 3), which is similar with Wang et al. (2016) and Liu et al. (2015). The main reason for the higher total residual soil ^15^N in Plastic compared with Open is a substantial reduction in N losses, especially the gas emission (Table 3). Most of the residual ^15^N was as non-mineral N forms for both Open and Plastic (Pilbeam et al., 2002). However, an average of 48.2% of residual total ^15^N was as mineral ^15^N in Plastic-Ridge, and the mineral ^15^N was 10–100 times larger than that for Plastic-Furrow or Open. This higher mineral N in Plastic-Ridge might be related to the following two reasons: 1) the ^15^N fertilizer was applied in the top soil layers and the plastic film prevented N leaching by rainfall (Jiang et al., 2018); 2) the increase in mineralization of microbial-assimilated organic N (the immobilized urea-^15^N by microorganisms at early stage of maize growing season) due to the higher temperature and moisture in mulched soil at late stage of maize growing season (Hai et al., 2015). Although both N mineralization and N immobilization processes occur simultaneously, a lower microbial biomass ^15^N and higher mineral ^15^N in Plastic (Fig. 4) implied that plastic film mulching might decrease the net immobilization of urea-^15^N in soil at harvest (Liu et al., 2015).

Three processes: ammonia volatilization, denitrification and N leaching are associated with the potential N losses in the urea-fertilized field. The N leaching was low both in Plastic and Open (Table 3). Nitrate is likely to accumulate in soil profile and occasionally leaches during heavy storms in this area (Zhou et al., 2016). There was only one heavy rainfall event larger than 40 mm in 2015. Hence, N leaching most likely accounted for only a small proportion of the N loss over the study period in Plastic and Open. In addition, the estimated N leaching was much lower in Plastic. Plastic film mulching reduces N leaching significantly compared to un-mulched soil (Ruidisch et al., 2013; Zhang et al., 2012; Wang et al., 2016b; Liu et al., 2015).

Plastic increased nitrous oxide (N_2_O) emission (meta-analysis of He et al., 2018), which was related to higher soil water content, nitrate concentration and soil organic carbon. However, the soil may also become a sink for N_2_O while under plastic mulch. Thus the studies on nitrous oxide under plastic film mulching are with contradictory results (Cuello et al., 2015; Kim et al., 2014; Liu et al., 2014; He et al., 2018), due to the difficulties to measure N_2_ losses from denitrification and to determine whether N_2_ release is influenced by mulching. However, due to the low precipitation in the study area, the loss of N fertilizer via denitrification, even in Plastic mulching, are unlikely to be high.

Ammonia volatilization is a major loss pathway of applied fertilizer N in the calcareous soil of the Loess Plateau, accounting for up to 50% of the applied urea-N (Roelcke et al., 1996), as soil pH is an important factor affecting ammonia volatilization **(**Sherlock et al., 1984, 1985**)**. Ammonia volatilization may be reduced with appropriate agricultural management, e.g. N fertilizer other than urea (e.g. KNO_3_) (Misselbrook et al., 2004). Mulching reduced N losses as ammonia emission by 30–64% (Shangguan et al., 2012; Liu et al., 2015). Compared with Open, Plastic had plastic film mulched ridge (accounting for 70% of total surface), likely resulting in lower ammonia volatilization and mainly explaining the lower N losses from Plastic.

### 4.3 Fate and transport of applied urea-N in Plastic-Ridge and Plastic-Furrow

N loss from the furrow, accounting for 78.6% of the total N losses in Plastic, were probably mostly caused by higher ammonia volatilization. The uncovered furrow may have been subject to high N loss by ammonia volatilization in the calcareous soil of Loess Plateau (Roelcke et al., 1996), which would be much reduced under the plastic film mulched ridge (Shangguan et al., 2012). Furrows are more prone to N leaching compared with ridges, due to higher water input and infiltration rates caused by the surface runoff from the ridges (Leistra and Boesten, 2010; Kettering et al., 2013). Although we observed higher N leaching from furrow (0.12 kg ha^−1^, compared with 0.03 kg ha^−1^ for the ridge), this value accounted for only a small part of N loss. More than 96% of the plant ^15^N uptake was derived from the Plastic-Ridge, with less than 4% from the Plastic-Furrow, implying that the ridge was the main source of fertilizer N for plant uptake and the furrow was the main source for N losses. A much higher residual soil ^15^N was found in Plastic-ridge, compared to Plastic-Furrow (Fig. 8). Except for the lower N loss from Plastic-ridge as discussed above, the another reason is the procedure used for fertilizer application, with approximately 70% of the applied fertilizer accumulating in the ridges during their creation. Therefore, we could reduce N losses and improve N use efficiency by decreasing the fertilizer N application rate to the furrow or with more precision placement of fertilizer N to the ridge only, or by using a form of N fertilizer other than urea (Ruidisch et al., 2013; Kettering et al., 2013).

The vertical distribution of total ^15^N and mineral ^15^N showed that N movement may be related to organic N released from root turnover and exudation in the maize root zone within the soil (Hodge et al., 2000), microbial-derived hydrophilic dissolved organic nitrogen (Kusliene et al., 2015), and nitrate leaching. Lateral movement of N was also observed and related to water movement (Fig. 6, Fig. 7). The pressure head gradients at the onset of rainfall were found to deviate horizontally in a water flow simulation under Plastic, indicating a lateral flow direction from the furrow to the ridge (Ruidisch et al., 2013a, b). However, the uneven soil water and heat conditions under mulching and plant water uptake may also cause a lateral flow direction from the ridge to the furrow or from the furrow to the ridge.

Mineral N in the top 0–20 cm soil decreased in both Plastic-Furrow and Open during the fallow period from October 2015 to April 2016. This is because nitrate leaching occurred at rainfall events during this period, which was confirmed by increased nitrate contents in 100–200 cm soil layer (measured before sowing in 2016, data not shown), compared to the data at harvest. However, the mineral N content increased in Plastic-Ridge during this period. This difference can be attributed to the practice of keeping the mulch after harvest. Mulching can reduce N leaching in the fallow period. Additionally, the decomposition of crop roots and rhizosphere microorganisms during the fallow period (Francois et al., 1991) resulted in a higher mineral N content in Plastic before sowing in 2016. Most of the fertilizer N not used by the crop in the season of application remained in the soil for potential use in the subsequent season.

### 4.4 Benefits of plastic film mulching

The maize yields were 12.9 and 14.2 Mg ha^−1^ for Open and Plastic, showing the high productivity in this area (Wang et al., 2016a). However, Plastic only increased yield by 9.7% due to the sufficient rainfall during maize growing season, especially the early stage (Fig. 1). Therefore, the net economic benefit was only 8.5% higher in Plastic than Open, which was much lower than the average benefit reported elsewhere (71.1%, Ma et al., 2018). Plastic film mulching usually increases grain yields significantly during drought years (Jiang et al., 2016) and thus the net economic benefit would be much more under drought conditions than that in our study.

Due to the high production, the crop uptake N was very high both in Open and Plastic. Other studies also found a higher crop uptake than N input (Liu et al., 2014; Haynes, 1999), showing high N use efficiency. However, the negative surplus N in soil and very high N use efficiency indicate soil mining of N (Table 4). This may be specific to the conditions of the cropping year for 2015 and/or may be a result of high soil fertility from a history of high fertilizer N input. However, continued soil N mining is unsustainable in the long term and appropriate N application rates should be recommended based on the target grain yield to maintain a sustainable farming system. Compared to Open, the Plastic reduced N losses and consequently benefited soil N retention. If the N loss could be further reduced in Plastic through appropriate N management strategies as stated above, the system N budget could be balanced. However, the higher mineral N in Plastic-ridge after harvest may indicate a high soil mineralization under mulched soil, which should be a topic of future studies.

## 5 Conclusions

Compared with Open, Plastic significantly increased maize grain yields by 9.7% in a semi-arid area. Total N uptake and uptake of applied urea-N were both increased under Plastic mulch. Estimated losses of the applied urea N via ammonium volatilization were very high, but lower under Plastic, and residual soil N up to 1.2 m depth was greater. Lateral movement of N from furrow to ridge and from ridge to furrow occurred in Plastic, facilitated by lateral movement of soil water. In Plastic, the ridges were the main source of fertilizer N uptake by the plants (> 96%), and the furrows were the main source of N losses (c. 79%). Briefly, Plastic increased yields and net economic benefit but changed the fate and transport of applied urea-N in maize production in semi-arid rain-fed croplands. Therefore, appropriate N management strategies should be designed for plastic mulching systems, such as minimal or zero fertilizer application to furrows or the use of N fertilizer forms other than urea (e.g. KNO_3_). This would improve N uptake and reduce gaseous N losses in the Plastic system to maintain the sustainability of agriculture in drylands.

## References

[bib0005] Abbasi M.K., Tahir M.M. (2012). Economizing nitrogen fertilizer in wheat through combinations with organic manures in Kashmir, Pakistan. Agron J.

[bib0010] Bhadauria N.S., Rajput R.L. (2015). Seed yield and nutrient uptake studies on clusterbean as influenced by mulching practices, varieties and nutrient management. Res. Crop..

[bib0015] Bremner J.M., Mulvaney C.S., Page A.L., Miller R.H., Keeney D.R. (1982). Nitrogen-total.

[bib0020] Brooks P.D., Stark J.M., Mcinteer B.B., Preston T. (1989). Diffusion method to prepare soil extracts for automated nitrogen-15 analysis. Soil Sci. Soc. Am. J..

[bib0025] Brookes P.C., Landman A., Pruden G., Jenkinson D.S. (1985). Chloroform fumigation and the release of soil nitrogen: a rapid direct extraction method to measure microbial biomass nitrogen in soil. Soil Biol. Biochem..

[bib0030] Bu L.D., Liu J.L., Zhu L., Luo S.S., Chen X.P., Li S.Q., Hill R.L., Zhao Y. (2013). The effects of mulching on maize growth, yield and water use in a semi-arid region. Agric. Water Manag..

[bib0035] Chakraborty D., Nagarajan S., Aggarwal P., Gupta V.K., Tomar R.K., Garg R.N., Sahoo R.N., Sarkar A., Chopra U.K., Sarma K.S.S., Kalra N. (2008). Effect of mulching on soil and plant water status, and the growth and yield of wheat (Triticum aestivum L.) in a semi-arid environment. Agric. Water Manag..

[bib0040] Cuello J.P., Hwang H.Y., Gutierrez J., Sang Y.K., Kim P.J. (2015). Impact of plastic film mulching on increasing greenhouse gas emissions in temperate upland soil during maize cultivation. Appl. Soil Ecol..

[bib0045] Dong H.Z., Li W.J., Tang W., Zhang D.M. (2009). Early plastic mulching increases stand establishment and lint yield of cotton in saline fields. Field Crops Res..

[bib0050] Francois C., Feller C., Guiraud G., Loury J., Boudot J.P. (1991). Immobilization of nitrogen from urea and plant residues in a ferrallitic soil: laboratory experiments and study by size-fractionation. Biol. Fertil. Soils.

[bib0055] Granlund K., Barlund I., Salo T., Esala M., Posch M. (2008). The effect of decreasing fertilization on agricultural nitrogen leaching: a model study. Agric. Food Sci..

[bib0060] Gan Y.T., Siddique K.H.M., Turner N.C., Li X.G., Niu J., Yang C., Liu L. (2013). Ridge-furrow mulching systems—an innovative technique for boosting crop productivity in semiarid rain-fed environments. Advan. Agron..

[bib0065] Hai L., Li X.G., Liu X., Jiang X.J., Guo R.Y., Jing G.B., Rengel Z., Li F.M. (2015). Plastic mulch stimulates nitrogen mineralization in urea-amended soils in a semiarid environment. Agron. J..

[bib0070] He G., Wang Z., Li S., Sukhdev S.M. (2018). Plastic mulch: tradeoffs between productivity and greenhouse gas emissions. J. Clean. Prod..

[bib0075] Hodge A., Robinson D., Fitter A. (2000). Are microorganisms more effective than plants at competing for nitrogen?. Trends Plant Sci..

[bib0080] Jenkinson D.S., Fox R.H., Rayner J.H. (2010). Interactions between fertilizer nitrogen and soil nitrogen—the so‐called ‘priming’ effect. Eur. J. Soil Sci..

[bib0085] Jiang R., Li X., Zhou M., Li H.J., Zhao Y., Yi J., Cui L.L., Li M., Zhang J.G., Qu D. (2016). Plastic film mulching on soil water and maize (Zea mays L.) yield in a ridge cultivation system on Loess Plateau of China. Soil Sci. Plant Nutr..

[bib0090] Jiang R., Li X., Zhu W., Wang K., Guo S., Misselbrook T., Hatano R. (2018). Effects of the ridge mulched system on soil water and inorganic nitrogen distribution in the Loess Plateau of China. Agric. Water Manag..

[bib0095] Kettering J., Ruidisch M., Gaviria C., Yong S.O., Kuzyakov Y. (2013). Fate of fertilizer ^15^N in intensive ridge cultivation with plastic mulching under a monsoon climate. Nutr. Cycl. Agroecosystems.

[bib0100] Kim Y., Berger S., Kettering J., Tenhunen J., Haas E., Kiese R. (2014). Simulation of N_2_O emissions and nitrate leaching from plastic mulch radish cultivation with LandscapeDNDC. Ecol. Res..

[bib0105] Kusliene G., Eriksen J., Rasmussen J. (2015). Leaching of dissolved organic and inorganic nitrogen from legume-based grasslands. Biol. Fertil. Soils.

[bib0110] Kuzyakov Y., Xu X. (2013). Tansley Review: Competition between roots and microorganisms for N: mechanisms and ecological relevance. New Phytol..

[bib0115] Kuzyakov Y., Friedel J.K., Stahr K. (2000). Review of mechanisms and quantification of priming effects. Soil Biol. Biochem..

[bib0120] Leistra M., JJTI Boesten (2010). Pesticide leaching from agricultural fields with ridges and furrows. Water Air Soil Pollut..

[bib0125] Li C.J., Li Y.Y., Yu CB Sun J.H., Christie P., An M., Zhang F.S., Li L. (2011). Crop nitrogen use and soil mineral nitrogen accumulation under different crop combinations and patterns of strip intercropping in northwest China. Plant Soil.

[bib0130] Liu J., Zhu L., Luo S., Bu L., Chen X., Yue S., Li S. (2014). Response of nitrous oxide emission to soil mulching and nitrogen fertilization in semi-arid farmland. Agric. Ecosyst. Environ..

[bib0135] Liu X.E., Li X.G., Guo R.Y., Kuzyakov Y., Li F.M. (2015). The effect of plastic mulch on the fate of urea-N in rain-fed maize production in a semiarid environment as assessed by ^15^N-labeling. Eur. J. Agron..

[bib0140] Ma D., Chen L., Qu H., Wang Y., Misselbrook T., Jiang R. (2018). Impacts of plastic film mulching on crop yields, soil water, nitrate, and organic carbon in Northwestern China: a meta-analysis. Agric. Water Manag.

[bib0145] Mbagwu J.S.C. (2010). Maize (Zea mays) response to nitrogen fertiliser on an ultisol in southern Nigeria under two tillage and mulch treatments. J. Sci. Food Agric..

[bib0150] Misselbrook T.H., Sutton M.A., Scholefield D. (2004). A simple process-based model for estimating ammonia emissions from agricultural land after fertilizer applications. Soil Use Manag..

[bib0155] Pilbeam C., Gregory P., Tripathi B., Munankarmy R. (2002). Fate of nitrogen-15-labeled fertilizer applied to maize-millet cropping systems in the mid-hills of Nepal. Biol. Fertil. Soils.

[bib0160] Ramakrishna A., Tam H.M., Wani S.P., Long T.P. (2006). Effect of mulch on soil temperature, moisture, weed infestation and yield of groundnut in northern Vietnam. Field Crops Res..

[bib0165] Rimski-Korsakov H., Rubio G., Lavado R.S. (2012). Fate of the nitrogen from fertilizers in field-grown maize. Nutr. Cycl. Agroecosystems.

[bib0170] Roelcke M., Han Y., Li S.X., Richter J. (1996). Laboratory measurements and simulations of ammonia volatilization from urea applied to calcareous Chinese loess soils. Plant Soil.

[bib0175] Romic D., Romic M., Borosic J., Poljak M. (2003). Mulching decreases nitrate leaching in bell pepper (Capsicum annuum, L.) cultivation. Agric. Water Manag..

[bib0180] Ruidisch M., Bartsch S., Kettering J., Huwe B., Frei S. (2013). The effect of fertilizer best management practices on nitrate leaching in a plastic mulched ridge cultivation system. Agric. Ecosyst. Environ..

[bib0185] Ruidisch M., Kettering J., Arnhold S., Huwe B. (2013). Modeling water flow in a plastic mulched ridge cultivation system on hillslopes affected by south korean summer monsoon. Agric. Water Manag..

[bib0190] Shangguan Y.X., Shi R.P., Li N., Han K., Li H.K., Wang L.Q. (2012). Factors influencing ammonia volatilization in a winter wheat field with plastic film mulched ridges and unmulched furrows. Huan Jing Ke Xue.

[bib0195] Sherlock R.R., Goh K.M. (1984). Dynamics of ammonia volatilization from simulated urine patches and aqueous urea applied to pasture I. Field experiments. Fertil. Res..

[bib0200] Sherlock R.R., Goh K.M. (1985). Dynamics of ammonia volatilization from simulated urine patches and aqueous urea applied to pasture. III. Field verification of a simplified model. Fertil. Res..

[bib0205] Wang C., Tian X., Li S. (2004). Effects of plastic sheet-mulching on ridge for rainwater-harvesting cultivation on WUE and yield of winter wheat. Scientia Agricultura Sinica.

[bib0210] Wang S., Luo S., Yue S., Shen Y., Li S. (2016). Fate of ^15^N fertilizer under different nitrogen split applications to plastic mulched maize in semiarid farmland. Nutr. Cycl. Agroecosystems.

[bib0215] Wang X., Xing Y. (2016). Effects of mulching and nitrogen on soil nitrate-N distribution, leaching and nitrogen use efficiency of maize (Zea mays L.). PLoS One.

[bib0220] Wang Y.P., Li X.G., Hai L., Siddique K.H.M., Gan Y., Li F.M. (2014). Film fully-mulched ridge-furrow cropping affects soil biochemical properties and maize nutrient uptake in a rainfed semi-arid environment. Soil Sci. Plant Nutr..

[bib0225] Wang Z.H., Zhang Y., Liu X.J., Tong Y.A., Qiao L., Lei X.Y. (2008). Dry and wet nitrogen deposition in agricultural soils in the Loess area. Acta Ecol. Sin..

[bib0230] Yang J., Gao W., Ren S. (2015). Long-term effects of combined application of chemical nitrogen with organic materials on crop yields, soil organic carbon and total nitrogen in fluvo-aquic soil. Soil Tillage Res..

[bib0235] Zegada-Lizarazu W., Berliner P.R. (2011). Inter-row mulch increase the water use efficiency of furrow-irrigated maize in an arid environment. J. Agron. Crop. Sci..

[bib0240] Zhang H., Liu Q., Yu X., Wu Y G.L.ü (2012). Effects of plastic mulch duration on nitrogen mineralization and leaching in peanut (Arachis hypogaea) cultivated land in the Yimeng Mountainous Area, China. Agric. Ecosyst. Environ.

[bib0245] Zhao H., Xiong Y.C., Li F.M., Wang R.Y., Qiang S.C., Yao T.F., Mo F. (2012). Plastic film mulch for half growing-season maximized WUE and yield of potato via moisture-temperature improvement in a semi-arid agroecosystem. Agric. Water Manag..

[bib0250] Zhou L., Jin S., Liu C., Xiong Y., Si J., Li X., Gan Y., Li F. (2012). Ridge-furrow and plastic-mulching tillage enhances maize–soil interactions: opportunities and challenges in a semiarid agroecosystem. Field Crops Res..

[bib0255] Zhou J., Gu B., Schlesinger W.H., Ju X. (2016). Significant accumulation of nitrate in Chinese semi-humid croplands. Sci. Rep..

